# Contribution of Genetics to Parkinson's Disease and Future Prospects

**DOI:** 10.14789/ejmj.JMJ24-0052-P

**Published:** 2025-04-03

**Authors:** MANABU FUNAYAMA

**Affiliations:** 1 Research Institute for Diseases of Old Age, Graduate School of Medicine, Juntendo University, Tokyo, Japan; 1 Research Institute for Diseases of Old Age, Graduate School of Medicine, Juntendo University, Tokyo, Japan; 2 Department of Neurology, Faculty of Medicine, Juntendo University, Tokyo, Japan; 2 Department of Neurology, Faculty of Medicine, Juntendo University, Tokyo, Japan; 3 International Collaborative Research Administration, Juntendo University, Tokyo, Japan; 3 International Collaborative Research Administration, Juntendo University, Tokyo, Japan

**Keywords:** Parkinson’s disease, genetics, risk, mutation

## Abstract

Parkinson’s disease (PD) is a systemic neurodegenerative disorder that is characterized by motor and non-motor symptoms. Although aging is the primary risk factor, environmental and genetic factors also contribute to risk, and identifying genetic risks may aid in preventive strategies. The present perspective outlines the two main genetic research strategies: research into familial PD using known causative gene screening and next-generation sequencing, and the analysis of sporadic PD using genome-wide association studies (GWAS). Recent advances in next-generation sequencing have improved gene screening, allowing researchers to quickly and inexpensively identify novel rare variants. However, challenges remain, such as accurately analyzing repetitive sequences and structural variants. The role of neurologists in gathering clinical and genomic data ─ especially from familial cases ─ is crucial. International collaborations, such as the Global Parkinson’s Genetic Program, address issues such as population diversity and missing heritability in GWAS. Contributions from Juntendo University include the discovery of PD-related genes and the implementation of validation studies in Japanese populations. We also aim to develop molecular targeted therapies using induced pluripotent stem cells. To elucidate the unknown causes of PD and advance treatment approaches, it is important to continuously conduct genetic research.

## Parkinson’s disease risk

Parkinson’s disease (PD) is a neurodegenerative disorder that presents with motor symptoms such as resting tremor, rigidity, dyskinesia, and postural instability. In addition, non-motor symptoms such as constipation, olfactory dysfunction, and rapid eye movement sleep behavior disorder are observed from early in the disease, or even before the appearance of parkinsonism. Furthermore, 30% to 80% of patients with PD develop dementia, such as memory loss and hallucinations, as the disease progresses^1)^. It is therefore important to understand PD as a systemic disorder, and not just as a movement disorder caused by neuronal degeneration in the midbrain.

The most important risk factor for developing PD is aging^2)^. Additionally, environmental factors and lifestyle habits gradually accumulate risk, and it is thought that the disease develops when the threshold is exceeded ─ just like a glass of water overflowing^3)^. In terms of the risks of disease development, we are born with genetic risk. Identifying the genetic risk of diseases permits individuals to predict which diseases they are most likely to develop in the future, allowing them to take measures to prevent disease onset.

## Strategies for identifying disease-related genes

**Figure 1:**
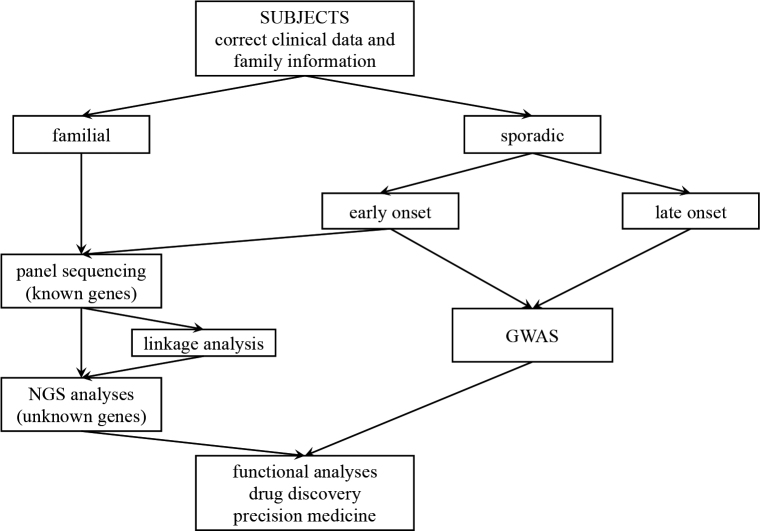
Strategies for the genetic analysis of patients with PD Subjects are first classified as familial or sporadic based on clinical and family information. Of the sporadic cases, patients with early onset are likely to have a large genetic factor in the disease development, and panel analysis is therefore performed. Our panel analysis covers the following genes: *SNCA, PRKN, Uch-L1, PINK1, DJ-1, LRRK2, ATP13A2, GIGYF2, HTRA2, PLA2G6, FBXO7, VPS35, EIF4G1, DNAJC6, SYNJ1, DNAJC13, CHCHD2, GCH1, NR4A2, VPS13, CRAB7L1, BST1, C19orf12, RAB39B, MAPT, PSEN1, GRN, APP, APOE, VPS13A, VPS13B, VPS13D, GBA, LRP10, UQCRC1, PSAP, ELOVL7, SMPD1, RIC3, NUS1, ARSA, TMEM175, PSEN2, GBA2, CTSD, ASAH1, LIN28A, DCTN1, WARS2, COMT, PI4KA, PTPA, COQ2, CTSB, ANXA1, ATP10B, BCKDK, GPNMB, MCCC1, MICU1, NRXN2, NUCKS1, PLXNA1, PODXL, PPP2R5D, PTPRA, PTRHD1, SORL1, SQSTM1, STK39, TENM4, TMEM229B, TMEM230, TRAP1, TWNK, UTS2, WASL, UBQLN2, UBQLN4, PSMF1, RAB32, DAGLB, STING1.* Familial PD cases are analyzed to search for new causative genes, and sporadic PD cases are analyzed to search for susceptibility genes.

According to the Online Mendelian Inheritance in Man (OMIM, https://omim.org/) database, approximately 7,500 genes have been reported as involved in human diseases. How are genes that are involved in the phenotype of a disease identified? Two major strategies are generally used to identify genes related to PD. The first is to screen for previously reported genes that cause familial PD with Mendelian inheritance. If no pathological variants in the known genes are identified, novel genes can be searched for using whole exon and/or whole genome sequencing using next-generation sequencing (NGS). Second, a genome-wide association study (GWAS) can be performed to determine the genetic risk of “sporadic” PD, which accounts for the majority of patients with the disease. Once PD-related genes and susceptibility genes are identified, the pathophysiology of the disease can be elucidated using cultured cells, induced pluripotent stem (iPS) cells, and animal models, with the goal of developing new drug targets and curative therapies (Figure 1).

## Important considerations in gene hunting

Genes associated with PD are numbered *PARK#*, and *PARK1* to 26 are currently registered (Table 1). More than 10 PD-related genes that have not been assigned *PARK* numbers have also been reported. In recent years, the discovery of genes related to PD has accelerated. This is because NGS technology has made it possible to quickly and inexpensively obtain large amounts of genetic sequencing data. NGS has also substantially refined the screening of known PD-related genes.

In 2016, our group developed a panel sequence for PD and dementia, and we have analyzed more than 2,800 subjects to date^4)^. In this panel sequencing, approximately 40% of subjects had rare variants in known PD-related genes. This is approximately double the positive rate of our previous results, which were obtained by screening only specific exons using Sanger sequencing. Thus, panel sequencing has made it possible to accurately and efficiently select families with familial PD of unknown causes. However, panel sequencing is not at all-powerful sequencing method. For example, short-read sequencing, including panel sequencing, is not particularly good at accurately aligning “repeat” sequences, which account for more than 50% of the human genome^5)^. Furthermore, structural variants and pseudogenes can cause genotyping difficulties.

Using long-read sequencing, our group has analyzed monozygotic twins who developed PD at the age of 17, who had already been found to have a monoallelic exonic deletion of exon 3 in *PRKN*. In this way, we identified a large inversion that included the *PRKN* region in the twins^6)^. The important take-home message from this discovery, in addition to recognizing the utility of long-read sequencing, is to carefully observe the phenotype of a subject, make a working hypothesis based on the clinical question, and choose the most effective analytical method.

The preconceptions of researchers can also sometimes make it difficult to identify causal gene variants. For example, our group previously reported two hereditary PD families with *LRRK2* c.4322G>A (p.Arg1441His)^7)^. We speculated that these two families were both of consanguineous marriage and autosomal recessive. However, the patients had a mixture of monoallelic and biallelic causal *LRRK2* variants. We first focused on variants that were homozygous in all patients, meaning that the causal *LRRK2* variant was ruled out, and it took a long time to identify the cause of PD in these families. Since *LRRK2* has already been reported to be the causative gene of autosomal dominant PD, it is known that patients with PD caused by *LRRK2* variants have monoallelic variants (Table 1). On the other hand, since *LRRK2* p.Gly2019Ser is the most frequent pathogenic variant of PD in Arabs and Ashkenazi Jews in North Africa and Caucasians in Europe and the United States, some PD patients with incidental biallelic p.Gly2019Ser have been reported. And PD patients with *LRRK2* p.Gly2019Ser have been reported to present a typical PD phenotype regardless of whether the variant is monoallelic or biallelic^8)^. Notably, the selection of appropriate research subjects and analytical methods ─ and the avoidance of human error, such as in the aforementioned examples ─ will probably be solved by artificial intelligence in the near future.

**Table 1:** OMIM-registered genes related to PD

Locus	Chromosomal region	Gene symbol
*PARK1/4*	4q21.3-q22	*SNCA*
*PARK2*	6q25.2-27	*PRKN*
*PARK3*	2p13	*unknown*
*PARK5*	4p13	*UCH-L1*
*PARK6*	1p36.12	*PINK1*
*PARK7*	1p36.23	*PARK7*
*PARK8*	12q12	*LRRK2*
*PARK9*	1p36	*ATP13A2*
*PARK10*	1p32	*unknown*
*PARK11*	2q36-q37	*GIGYF2*
*PARK12*	Xq21-q25	*unknown*
*PARK13*	2p13.1	*HTRA2*
*PARK14*	22q13.1	*PLA2G6*
*PARK15*	22q11.2-qter	*FBXO7*
*PARK16*	1q32	*unknown*
*PARK17*	16q12	*VPS35*
*PARK18*	3q27-qter	*EIF4G1*
*PARK19*	1p31.3	*DNAJC6*
*PARK20*	21q22.11	*SYNJ1*
*PARK21*	3q22.1	*DNAJC13*
*PARK22*	7p11.2	*CHCHD2*
*PARK23*	15q22.2	*VPS13C*
*PARK24*	10q22.1	*PSAP*
*PARK25*	9q34.11	*PTPA*
*PARK26*	6q24.3	*RAB32*

OMIM, Online Mendelian Inheritance in Man. *PARK1* was reported as a single nucleotide variant, and *PARK4* as a copy number variant, of *SNCA*.

## Contributions of neurologists to familial PD research

Because most cases of familial PD are inherited in a Mendelian fashion, it is important to collect as much genomic data as possible, from both the affected and unaffected individuals within a family. Whole exome sequencing usually detects several hundred variants per subject. If the disease-causing variant is among these variants, then the “true” causal variant needs to be identified. If you only have the genomic data of the founder, finding the “true” variant is more difficult than finding a ring dropped on the beach. If the disease-causing variant is de novo, it may be possible to find the causative variant by performing a trio analysis of the asymptomatic carrier parents and the child with the disease. However, identifying a causative variant in late-onset PD is more difficult. This is because, in addition to the causative variant, there are also complex interactions among age-related risks and environmental factors, making it difficult to determine whether unaffected subjects also have the causative variant and whether or not they will develop the disease. It is therefore important to estimate the penetrance rate by obtaining information about the disease onset from as many family members as possible, in addition to collecting genomic data. Performing linkage analysis by combining clinical and genomic information in this way is very useful for identifying “true” causal variants. Thus, although technological innovation has increased the speed of genomic analysis by several dozen times, the efforts of physicians in collecting data on patients and family members are still very important. We must remember that the genetic research of diseases would not be possible without the contributions of physicians, who discover valuable cases through daily patient examinations, and who sometimes travel by plane to collect genomic and clinical information from family members.

## Importance of international collaborative research in GWAS

In contrast to the analysis of small-scale familial disease pedigrees, GWAS is a method for analyzing case-control subjects on a large scale to identify genetic risk. The main aim of GWAS is to compare the genetic variants of a group of people with the same disease and a control group, to identify genetic variants with different allele frequencies. These genetic variants are then considered to be related to the disease. GWAS is a data-driven method, and it can only be used to determine the chromosomal location information of the related genes. GWAS mainly uses microarray technology; microarrays can determine hundreds of thousands to millions of genotypes using chips the size of a glass slide. Although microarrays have the advantage of being inexpensive per subject, they also have the disadvantage of only being able to determine a limited number of genetic variants.

Recently, the number of GWAS studies using whole genome analysis data has increased; however, a certain level of capital investment is required because of the large amounts of data that need handling. Moreover, there are several problems with GWAS research. First, it is necessary to collect data from a large number of subjects, ranging from tens of thousands to hundreds of thousands. Second, more than half of the GWAS studies published to date have been conducted on European populations^9)^. Third, there is the problem of missing heritability^10)^, which refers to findings indicating that the heritability identified in familial and monozygotic twin studies is larger than that identified in GWAS. Polygenic risk scores and genotype imputation are two methods for solving the problem of missing heritability; however, these are greatly affected by racial differences. Large-scale international collaborative research is therefore essential to tackle these problems in GWAS research.

The Global Parkinson’s Genetic Program (GP2) is one of the largest international collaborative studies of PD genetics^11)^. One recent result of the GP2 project is that *GBA1*, which was not a hit in the European PD GWAS study, was a hit in the African PD GWAS study^12)^. GP2 is one of the resource projects of Aligning Science Across Parkinson’s (ASAP, https://parkinsonsroadmap.org). ASAP is supported by a long-term commitment from the Sergey Brin Family Foundation ─ the personal foundation of Sergey Brin, co-founder of Google. Sergey Brin announced on his blog in 2008 that he has the *LRRK2* p.Gly2019Ser variant, which is most common in Ashkenazi Jews (approximately 14%) and North African Berbers (30%-40%)^13)^. For this reason, Sergey Brin continues to provide much financial support to help overcome PD. The original *LRRK2* family is the “Sagamihara family” in Sagamihara City, Kanagawa Prefecture, Japan^14)^. The “Sagamihara family” was published as a Japanese- language paper in 1978^15)^. It is very interesting that a 45-year-old case report in Japanese has become a research topic that now involves hundreds of thousands of researchers and physicians, as well as hundreds of thousands of subjects.

## Contributions of Juntendo University to PD genetics

At Juntendo University, we have been conducting research on PD genetics for over 25 years. We have discovered three genes that cause hereditary PD: *PARK2*, *PARK22*, and *PARK24*^16-18)^. We have also conducted in large-scale validation studies of PD-causing genes such as *PRKN*, *PINK1*, *PARK7*, *LRRK2*, and *VPS35* in Japanese patients^19-23)^. In addition, we have reported rare cases with *SNCA*, *ATP13A2*, *FBXO7*, and *PLA2G6* variants^24-28)^. Notably, we have also succeeded in establishing iPS cells from patients with these familial PD-causing variants, and we are currently aiming to better elucidate the pathogenesis of the disease and develop molecularly targeted therapeutic drugs^29)^.

## Conclusions

In this Perspectives, I have outlined the genetics of PD as well as strategies for its genetic analysis. In addition, I have described my experience and approach to research that has led to new discoveries. The contributions of neurologists, the importance of international collaborative research, and the contributions of Juntendo University to molecular genetic research on PD were also introduced. Approximately 60% of patients with familial PD still have an unknown cause, and it is expected that many new genes related to PD onset will be discovered in the future. I hope that this Perspectives will be of use to researchers, neurologists, and patients who are tackling molecular genetic research.

## Funding

This work was supported by the Japan Society for the Promotion of Science (JSPS) KAKENHI [Grant Number: 24K02372 for MF]; and from the Research Institute for Diseases of Old Age, Juntendo University Graduate School of Medicine.

## Author contributions

MF: Manuscript writing of the first draft, and review.

## Conflicts of interest statement

The author declare that there are no conflicts of interest.
